# The Association between Tooth Loss and Alzheimer’s Disease: a Systematic Review with Meta-Analysis of Case Control Studies

**DOI:** 10.3390/dj7020049

**Published:** 2019-05-01

**Authors:** Mario Dioguardi, Giovanni Di Gioia, Giorgia Apollonia Caloro, Giorgia Capocasale, Khrystyna Zhurakivska, Giuseppe Troiano, Lucio Lo Russo, Lorenzo Lo Muzio

**Affiliations:** 1 Department of Clinical and Experimental Medicine, University of Foggia, Via Rovelli 50, 71122 Foggia, Italy; digioia-giovanni@outlook.it (G.D.G.); khrystyna.zhurakivska@unifg.it (K.Z.); giuseppe.troiano@unifg.it (G.T.); lucio.lorusso@unifg.it (L.L.R.); lorenzo.lomuzio@unifg.it (L.L.M.); 2 Department of Emergency and Organ Transplantation, Nephrology, Dialysis and Transplantation Unit, University of Bari Via Piazza Giulio Cesare, 70124 Bari, Italy; giorgiacaloro1983@hotmail.it; 3 Department of Surgical, Oncological and Surgery, University of Palermo, 90121 Palermo, Italy; giorgia.capocasale@unipa.it

**Keywords:** Alzheimer’s disease, dementia, periodontitis, tooth loss

## Abstract

Alzheimer’s disease is classified as a neurodegenerative condition, a heterogeneous group of illnesses characterized by the slow and progressive loss of one or more functions of the nervous system. Its incidence tends to increase gradually from 65 years of age, up to a prevalence of 4% at age 75. The loss of dental elements is more prevalent in this population and might negatively affect the masticatory capacity, quality of life, and pathogenesis of Alzheimer’s disease. This study investigated problems related to oral health and the loss of dental elements in elderly patients suffering from Alzheimer’s and considered whether local inflammatory processes could affect the etiopathogenesis of Alzheimer’s disease. The purpose of this systematic review is to identify a link between the causes leading to tooth loss and the onset/progression of Alzheimer’s disease. We also studied whether there is a higher incidence of tooth loss (primary outcome) and edentulism (secondary outcome) among Alzheimer’s patients. We searched records in electronic databases such as PubMed, EBSCO, and Web of Science using the following keywords: Alzheimer’s Disease AND periodontal, Alzheimer’s Disease AND periodontitis, dementia AND (periodontitis OR periodontal) “Alzheimer’s Disease” AND “tooth” OR “dental loss,” “dementia” AND “edentulous,” “Alzheimer’s Disease” AND “edentulous,” “dementia” AND “tooth” OR “dental loss.” The records were screened, and after applying the eligibility and inclusion criteria, nine articles were left, six of which were analyzed for the primary outcome (loss of dental elements) and six for the secondary outcome (tooth loss). Results from this meta-analysis revealed that Alzheimer’s disease patients have an increased risk of dental loss (hazard ratio (HR) 1.52, 95% confidence interval (CI) 1.00–2.30, *p* = 0.05) and edentulous condition (HR 2.26, 95% CI 1.70–3.01, *p* < 0.001). A quantitative analysis of the included studies indicated that patients suffering from Alzheimer’s disease are characterized by a greater number of lost dental elements and general edentulism compared to the control groups.

## 1. Introduction

Diseases related to aging in Western countries are becoming increasingly prevalent with a more significant socioeconomic impact due to the increase in the average lifespan. One such condition, Alzheimer’s disease (AD), is characterized by the slow and progressive loss of one or more functions of the nervous system [1]. A recurring sign of this pathology is dementia, defined as “a significant decrease or total loss of intellectual functions, which gradually and progressively takes place, due to different causes in previously healthy subjects [2].” The elderly need to be able to face senility under improved conditions through a reduction in the most disabling manifestations of neurodegenerative diseases, such as dementia associated with Alzheimer’s disease [3].

One of the main causes of tooth loss is periodontal disease. Periodontitis is an oral pathology that affects the dental support apparatus, leading to the destruction of the soft tissues and to the resorption of the alveolar bone up to the loss of the dental elements [4]. Periodontitis is a pathology with a bacterial etiology that affects 50% of the world’s population [5] and presents risk factors such as poor oral hygiene [6], smoking [7] and alcohol consumption [8].

Tooth loss can negatively influence cognitive function in various ways, one of which is inflammation induced by periodontal disease. Periodontitis is reported as one of the main factors that cause tooth loss and is linked to an augmentation of interleukin-1 (IL-1), tumor necrosis factor-α (TNF-α), and IL-6, which are well-known pro-inflammatory agents. Their increased levels in plasma leads to inflammatory processes affecting the brain, contributing to cognitive decline [9]. This is relevant to Alzheimer’s disease because classic theory describes histopathological alterations caused by senile plaques, with neurofibrillary tangles and neuronal losses that are secondary to the deposition of β-amyloid substances due to altered metabolism of amyloid precursor protein (APP). An inflammatory theory has also been put forward, in which peripheral inflammatory processes trigger the precipitation of β-amyloid [10]. This β-amyloid precipitation can be combined with local subacute (central nervous system, CNS) and peripheral inflammation, sustained mainly by Gram-negative bacteria, such as those that act as etiologic agents for periodontal [11,12] and endodontic [13,14,15] lesions.

In addition, tooth loss results in masticatory dysfunction, which leads to poorer nutrition and subsequent reduction in cerebral blood perfusion [16]. A decrease in acetylcholine levels and the number of pyramidal cells involving the hippocampus have been observed in different models, highlighting a link with masticatory impairment [17].

Another aspect to keep in mind is the difficulty of dental rehabilitation in subjects suffering from dementia. A study conducted by Faggion C.M. et al of 2013 reports [18], in a systematic review of the literature, a higher incidence of peri-implantitis cases in patients with Dementia and other neuro-degenerative diseases, according to Faggion C.M. the patient suffering from Alzheimer’s disease may have an improvement in the masticatory component from the use of prothesis loaded on implants (overdenture). Furthermore, he adds that the periimplantitis problem can be kept under control by sensitizing the health professionals assigned to assist the patient.

### Rational Meta Analysis

The loss of teeth in the elderly population represents a problem for their general health, as the reduced masticatory capacity can lead to an unsuitable diet. Moreover, if the patient is suffering from dementia due to Alzheimer’s disease, the maneuvers related to oral hygiene and the prevention of periodontal disease can be reduced, causing a greater loss of dental elements. More studies find links between the pathogenesis of periodontal disease and neuro-degeneration in Alzheimer’s disease [19], finding common features in local inflammatory conditions [20]. So in light of this, tooth loss, especially if induced by periodontitis, may represent an additional problem for patients with Alzheimer’s disease.

The scientific literature agrees in saying that the loss of dental elements can represent a problem in elderly subjects much more that are suffering from Alzheimer’s disease. In fact, a study conducted by Aragón F. et al 2017 concludes that Alzheimer’s patients had a worse oral health (caries and periodontal disease) with a greater amount of oral lesions to the mucosa, with a qualitative and quantitative deterioration of the saliva [19]. In addition to these considerations, another longitudinal study conducted by Fereshtehnejad SM et al. 2018 indicates how the use of dental care decreases considerably after the diagnosis of dementia, and this reduction is more marked in those with rapid cognitive impairment [21].

As previously mentioned, the probable causes of the loss of dental elements can be indicated in the elderly suffering from dementia by a gradual abandonment of the oral hygiene maneuvers and by the abandonment of dental care [22].

Our question is to establish whether there is a greater incidence of loss dental elements in Alzheimer’s disease compared to a population of similar age in case control studies. Previous systematic reviews have evaluated both soft tissue lesion and the presence of caries and loss elements in cohort, longitudinal and case control studies but without performing a quantitative analysis [12,23], furthermore, this paper is currently the only research on the topic that includes a case control study for the 2 outcomes in the meta-analysis.

This revision can be seen through statistical analysis and an association between two events: the loss of the dental elements/edentulism, and the Alzheimer’s disease giving more elements to the doctor and health professionals in the prevention of the periodontal problems that lead to the loss of dental elements, in light of the possible associations between local inflammation and the onset and progression of Alzheimer’s disease.

The aim of this systematic review and meta-analysis is to highlight the possible association between dental loss and Alzheimer’s disease in order to elaborate on the multifactorial background of this disease and patients’ general health status [24,25].

## 2. Materials and Methods

The following review was performed based on indications from the PRISMA protocol [26]. After an initial screening phase, eligible publications were studied by qualitative analysis and by searching for “outcomes” from which to draw linking hypotheses or new indications for future research.

### 2.1. Eligibility Criteria

The works taken into consideration were epidemiological research studies, literature reviews and clinical studies, with greater attention being paid to case control studies focused on the links between oral health, specifically periodontal and oral diseases, and dementia associated with Alzheimer’s disease, conducted within the last 30 years and published in English.

### 2.2. Research Methodology

The studies were identified through bibliographic searches in electronic databases and by examining the bibliographies of published articles. Bibliographic research was conducted in search engines including PubMed, EBSCO, and Web of Science between 11 February 2018 and 10 March 2018, and the last search for a partial literature update was performed on 4 October 2018.

The following search terms or combinations of them were used: Alzheimer’s disease AND periodontal (109, 131, and 117 records for PubMed, EBSCO, and Web of Science, respectively); Alzheimer’s disease AND periodontitis (86, 103, and 84 records); dementia AND (periodontitis OR periodontal) (163, 180, and 185 records); “Alzheimer’s Disease” AND “tooth” OR “dental loss” (186, 254, and 192 records) “dementia” AND “edentulous” (40, 51, and 28 records); “Alzheimer’s Disease” AND “edentulous”(7, 9, and 3 records); “dementia” AND “tooth” OR “dental loss” (232, 359, and 246 records).

The results are summarized in Table 1.

For systematic reviews and reviews of clinical trials and clinical studies, the restrictions were applied for each “keyword” in order to search for previous systematic reviews and to investigate possible outcomes.

### 2.3. Screening Methodology

The search for and subsequent screening of records was conducted by two independent reviewers. A third reviewer had the task of deciding in uncertain situations. Screening included analyzing the titles and abstracts to eliminate records that were not relevant to the issues addressed in the review; “overlaps” were then discarded. Finally, potentially eligible articles were submitted to a full-text analysis for verification via qualitative analysis; a fourth reviewer resolved disputes.

Eligible studies were those that determined a correlation between dementia in the course of Alzheimer’s and oral health; as criteria for inclusion in the meta-analysis, clinical case control studies that presented data regarding the loss or presence of teeth and edentulism were taken into consideration for both groups.

Two reviewers were responsible for research and screening: M.D., a PhD and 1 year postdoc, and G.Di G., a postgraduate. The third reviewer was G.T., a PhD and DDS, and all three of these junior researchers were based at the Department of Clinical and Experimental Medicine at the University of Foggia (Italy). The fourth reviewer, who performed the supervisory tasks, was L.Lo.M., a DDS and MD, director of the Department of Clinical and Experimental Medicine.

## 3. Results

**Figure 1:**
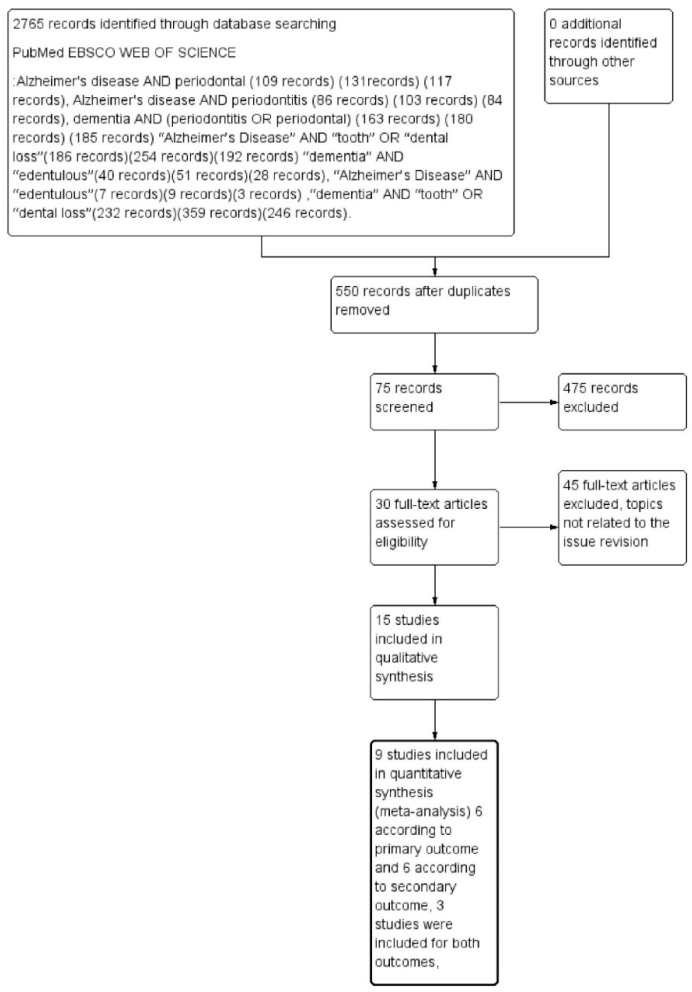
Flowchart describing research methodology, following the PRISMA protocol guidelines.

The outcomes selected were loss of dental elements (primary outcome) and total edentulism (secondary outcome). A total of 2765 records were identified on PubMed, EBSCO, and Web of Science databases. After deleting the remaining overlapping records, we obtained a total of 550 records. Applying the eligibility criteria, 75 articles were read as full text, and only nine articles were eligible for inclusion. These were evaluated for quantitative assessment—six related to the primary outcome and six related to the secondary outcome, with three studies included for both outcomes, as described in Figure 1.

**Figure 2:**
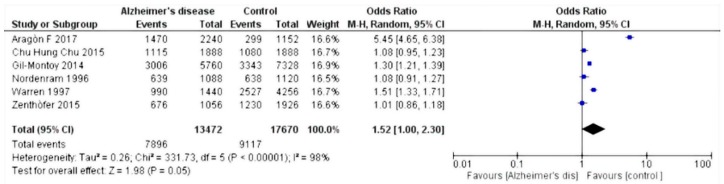
Forest plot of random effects models of the meta-analysis. Outcome: missing teeth.

**Figure 3:**
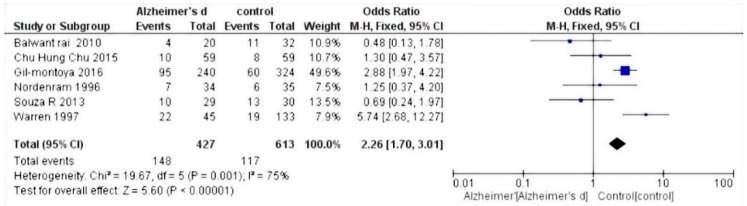
Forest plot of fixed effects models of the meta-analysis. Outcome: edentulism.

The selected studies were those by Chun et al. 2015 [27]; Warren et al. 1997 [28]; Gil-Montoya et al. 2014 [29]; Zenthòfer et al. 2015 [30]; Aragòn et al. 2018 [31]; Nordenram et al. 1996 [32]; Gil-Montoya et al. 2016 [33]; Rai et al. 2010 [34]; and De Souza Rolim et al. 2013 [35]. Results of the meta-analysis based on the six included studies showed an increased risk of dental loss (hazard ratio (HR) 1.52, 95% confidence interval (CI) 1.00–2.30, *p* = 0,05) (Figure 2), whereas based on the five relevant studies, Alzheimer’s disease patients have an increased risk of experiencing edentulous conditions (HR 2.26, 95% CI 1.70–3.01, *p* < 0.001) (Figure 3).

### Study Characteristics and Data Extraction

The characteristics of the selected studies are described in Table 2 (primary outcome, missing teeth) and Table 3 (secondary outcome, edentulism) with the relevant data extrapolated.

The studies chosen were all case controls, and the risk of bias was assessed using the Newcastle–Ottawa scale for case control (Table 4).

Data were extracted by two reviewers and are reported in two tables. The extracted data were the total number of missing dental elements (primary outcome) and the number of patients with edentulism (secondary outcome) in the respective control and Alzheimer’s disease groups.

## 4. Data Analysis

Statistical analysis of the data was performed using Rev Manager 5.3 software (Cochrane collaboration, Copenhagen, Denmark) and illustrated using forest plot charts for the two outcomes. The first outcome was the loss of dental elements. From the meta-analysis, heterogeneity was revealed when calculating the odds ratio, with the value of I^2^ equal to 98%; thus, a random effects model was applied.

Since the outcome was negative, the results of the forest plots were in favor of the controls. For the secondary outcome, i.e., the number of people with edentulism, a fixed effects model was applied. As the heterogeneity was equal to an I^2^ value of 75%, the result was in favor of controls compared to those affected by Alzheimer’s disease.

## 5. Discussion

The results show that in patients with Alzheimer’s, there is a greater loss of teeth and cases of edentulism than in subjects who do not have the disease. For elderly people, the loss of dental elements, whether due to causes related to carious processes or related to periodontal or endodontic problems, leads to a decrease in masticatory capacity and quality of life [36,37]. 

The local inflammatory responses have been increasingly placed in a correlation with the onset of Alzheimer’s disease, furthermore a further contribution in the progression of the disease could come from the reduction to the attention to the oral hygiene of the patients. Since patients with Alzheimer’s a fortiori have difficulty with mnemonic skills, they gradually reduce their attention to oral hygiene issues, which can result in deterioration of their oral health [23], leading to peripheral inflammatory processes that may contribute to the onset and progression of Alzheimer’s disease [38]. An epidemiological correlation between AD pathogenesis and the loss of dental elements is supported by different cohort studies. In particular, Takeuchi et al. showed a correlation between dental loss and dementia, with an inverse association between the number of residual dental elements and the risk of developing dementia, particularly AD. This association was not shown for dementia of vascular origin. Takeuchi et al. stressed three main mechanisms [24]. First of all, the presence of the teeth, and therefore of the masticatory capacity, increases cerebral blood flow, improving oxygenation in the cortical area, with an evident protective effect against the onset and progression of the disease.

Second, loss of dental elements can lead to dietary changes, with a loss of nutrition being significant for the body as it can lead to the risk of malnutrition, which promotes the onset of dementia [39]. Finally, the chronic systemic inflammation that is taken into consideration in the etiopathogenesis of Alzheimer’s disease finds further support from the local inflammatory status determined by periodontitis, which is the main cause of the loss of dental elements in adults. Okamoto et al. reported similar results, highlighting how endotoxins and cytokines can cross the blood–brain barrier [40]. This mechanism is also supported by common genetic alterations existing in both periodontitis and AD, such as interenzyme polymorphisms for IL-1A and IL-1B [41,42]. 

The majority of studies reviewed in this paper are in agreement with the reduction of oral hygiene in patients affected by Alzheimer’s disease, with a significant worsening of their oral health status. Results from this meta-analysis show that Alzheimer’s disease patients have increased risks for dental loss (HR 1.52, 95% CI 1.00–2.30, *p* = 0.05) and edentulous conditions (HR 2.26, 95% CI 1.70–3.01, *p* < 0.001).

These results should be interpreted with consideration of the risk of bias between studies. In particular, points of weakness were highlighted with the Newcastle–Ottawa scale, with poor quality of evidence emerging between studies. All of the included studies were case controls; studies on randomized controlled trials were not eligible for inclusion because of the missing outcome of the dental status. In addition, high heterogeneity emerged between the included studies. For example, all of the studies included patients of different nationalities. It is well known that genetic differences among races could influence predisposition to some diseases, as could traditional and social conditions that differ among cultures [43,44,45,46]. In addition, sample size is a limiting factor, which increases the heterogeneity not only between the included studies, but also within the study itself, with a differential statistical power between the control and test groups. For these reasons, future studies should consider standard inclusion criteria and comparability between groups. For example, in the Aragon paper [17], there is an age bias between Alzheimer’s disease patients (77.4 ± 10.6 years) and the control group (62.6 ± 7.1 years).

Another point of weakness concerns the clinical outcomes measured, such as the loss of teeth, which could occur from different causes, such as trauma, decay, or periodontitis. For these reasons, future research should investigate these variables, also including information from the salivary gland function, periodontal status, and which dental elements are mainly involved.

In particular, there is a loss of many dental elements, highlighting a deficit in patients taking care of themselves, that could be also influenced by general cognitive impairment and not specifically linked to Alzheimer’s disease.

## 6. Conclusions

We investigated the association between Alzheimer’s disease and dental loss. A quantitative analysis of the included studies indicated that patients suffering from Alzheimer’s disease are characterized by a greater number of lost dental elements and general edentulism compared to the control groups. Current evidence supports the concept that different biological mechanisms could be involved in dental loss, leading to the onset and progression of AD. The results can be summarized by seven main points:

1. Periodontitis, characterized by local inflammation and bacterial invasion, is one of the main causes of dental loss in adults. Local chronic inflammation could lead to the precipitation of β-amyloid (supported by a subacute inflammatory process at the CNS level). Furthermore, it is well known that different species of bacteria, endotoxins, and cytokines can reach the CNS.
2. Dental elements are important in the masticatory process; good masticatory capacity contributes to increased cerebral blood flow with increased oxygenation, and therefore has a protective role.
3. Dental loss also leads to decreased quality of feeding with decreased intake of vitamins and nutrients, which are important for the health of the CNS [47].
4. AD and periodontitis (one of the main causes of dental loss) share common genetic factors, for example, polymorphism of interleukin 1A-B [48].
5. Dental elements are also involved in proprioception, and an association between their loss and a decreased number of pyramidal cells in the hippocampus has been shown in a mouse model [49].

It is becoming more and more apparent that oral health is linked to general health status. Future medical practitioners should focus their attention on preventing diseases that could be linked with bad oral health and, in general, to tooth loss and periodontitis. Older patients usually experience cognitive decline and pay less attention to their oral hygiene. For these reasons, patients should take interest in preventive actions, and when needed, family/sanitary support should aid in this regard. For patients with an early diagnosis of Alzheimer’s disease who are not affected by cognitive impairment, it has been shown that prosthetic rehabilitation can improve their prognosis [50].

## Figures and Tables

**Table 1:** Search table summarizing records from PubMed, Web of Science, and EBSCO.

Keywords	PubMed	EBSCO	Web of Science	Total
Alzheimer’s disease AND periodontal	109 records	131 records	117 records	357 records
Alzheimer’s disease AND periodontitis	86 records	103 records	84 records	273 records
dementia AND (periodontitis OR periodontal)	163 records	180 records	185 records	528 records
“Alzheimer’s Disease” AND “tooth” OR “dental loss”	186 records	254 records	192 records	632 records
“dementia” AND “edentulous”	40 records	51 records	28 records	119 records
“Alzheimer’s Disease” AND “edentulous”	7 records	9 records	3 records	19 records
“dementia” AND “tooth” OR “dental loss”	232 records	359 records	246 records	837 records
Total	823 records	1087 records	855 records	2765 records

**Table 2:** Extracted data from selected studies (primary outcome, missing teeth). AD: Alzheimer’s disease.

Case Control, Author, Date	Dementia Group (AD)	Control Group	Missing Teeth AD	Missing Teeth Control
Chun et al. 2015	59	59	1115	1080
Warren et al. 1997	45	133	990	2527
Gil-Montoya et al. 2014	180	229	3006	3343
Zenthòfer et al. 2015	33	60	676	1230
Aragòn et al. 2018	70	36	1470	299
Nordenram et al. 1996	34	35	639	638

**Table 3:** Extracted data from selected studies (secondary outcome, edentulism).

Case Control, Author Date	Dementia Group (AD)	Control Group	Edentulous AD	Edentulous Control
Chun et al. 2015	59	59	10	8
Warren et al. 1997	45	133	18	42
De Souza Rolim et al. 2013	29	30	10	13
Nordenram et al. 1996	34	35	7	6
Gil-Montoya et al. 2016	240	324	95	60
Rai et al. 2010	20	32	4	11

**Table 4:** Assessment of risk of bias within the studies (Newcastle–Ottawa scale) with scores. (4–5 scores: Low quality, 6–7 scores: Intermediate quality, 8–9 scores: High quality).

	Selection				Comparability			Exposure		Score
Case Control Study	Definition of Cases	Representativeness of Cases	Selection of Controls	Definition of Controls	Age	Gender	Assessment of Oral Health	Same Method Cases and Controls	Nonresponse Rate	Total
Chun et al. 2015	−	+	−	+	+	+	+	−	−	5
Warren et al. 1997	+	+	−	−	+	+	+	+	+	7
Gil-Montoya et al. 2014	+	−	+	−	+	+	+	+	+	7
Zenthòfer et al. 2015	−	−	+	−	+	−	+	+	−	4
Aragòn et al. 2018	+	+	−	+	−	+	+	+	−	6
Nordenram et al. 1996	+	+	+	+	+	+	+	+	−	8
Rai et al. 2010	+	−	−	−	+	+	+	+	−	5
Gil-Montoya et al. 2016	+	−	+	−	+	+	+	+	−	6
De Souza Rolim et al. 2013	+	+	+	+	+	+	+	+	−	8
